# Flexible Conducting Composite Film with Reversible In‐Plane Folding–Unfolding Property

**DOI:** 10.1002/advs.202102314

**Published:** 2021-08-13

**Authors:** Peiru Sun, Chuao Ma, Yong Chen, Hongliang Liu

**Affiliations:** ^1^ School of Chemistry and Chemical Engineering Yantai University Yantai 264005 P. R. China; ^2^ School of Metallurgy and Materials Engineering Chongqing University of Science and Technology Chongqing 401331 P. R. China; ^3^ College of Chemistry Jilin University Changchun 130012 P. R. China

**Keywords:** flexible conducting films, in‐plane folding‐unfolding, reversible deformation, soft electronics

## Abstract

Flexible conducting films in the forms of bendability or stretchability are developed as a key component to enable soft electronics. With the requirements of miniaturization and portability of modern electronics, conducting film that can endure in‐plane shrinkage is urgently needed but still remains challenging. Here, a new type of conducting film achieving reversible in‐plane folding–unfolding function with large deformation by infusing conductive liquids into hierarchically structured polymer films consisting of both nanostructured polymer nanofibers and microstructured frames is reported. Nanostructured polymer nanofibers that can be completely wetted by the conductive liquids provide capillary forces to gain reversible in‐plane folding–unfolding property, while the microstructured frames greatly enhance the extent during folding–unfolding process. Conductivity of the produced films can be significantly improved by rationally tuning the composition of infused conductive liquids, which always keeps high values during the folding–unfolding deformation. It is believed that this work may serve as the basis for robust fabrication of flexible conducting films with reversible in‐plane folding–unfolding function, and can also put one‐step forward of modern soft electronics.

## Graphical abstract

The concept of “reversible in‐plane large deformation of conductive films” for next‐generation soft electronics is demonstrated. The in‐plane folding–unfolding performance is achieved by the combination of nanostructure‐induced capillary forces and microstructure‐assisted enhanced deformation. It is believed that this work provides a new way to advance the development of novel functional materials to satisfy the needs of modern soft electronics.

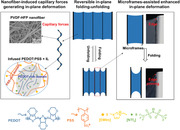

## 1 Introduction

Flexible conducting films, either electronically or ionically, are the underpinning of modern soft electronics and have been the subject of extensive studies including optoelectronic devices,^[^ ^1^ ^]^ electronic skin,^[^ ^2^ ^]^ wearable sensors,^[^ ^3^ ^]^ and stretchable actuators.^[^ ^4^ ^]^ Thanks to the discovery of conducting polymers,^[^ ^5^ ^]^ flexible polyaniline film was used for bendable light‐emitting diodes as early as 1992.^[^ ^6^ ^]^ After more than one‐decade continuous efforts, the conducting materials were extended to carbon nanotubes,^[^ ^7^ ^]^ graphene,^[^ ^8^ ^]^ metal grids or nanowires,^[^ ^9^ ^]^ and showed great successes in preparation of various bendable optoelectronic devices. Just after the turn of 2010s, stretchable conducting hydrogels,^[^ ^10^ ^]^ ionogels,^[^ ^11^ ^]^ and even organohydrogels^[^ ^12^ ^]^ were developed to satisfy the needs of stretchable electronics. For example, by using a conductive stretchable polyacrylamide hydrogel containing NaCl as the electrolyte, a large‐strain actuator was made for demonstration of a loudspeaker producing sound over the entire audible range.^[^ ^10b^ ^]^ Owing to the excellent stability of ionogels, soft devices that can function well under harsh conditions were also designed by using stretchable ionogels as the conductive components.^[^ ^11e,g,h^ ^]^ The success of bendable and stretchable conducting films as key components for soft electronics has remained, but a question arises: can conducting films also be harnessed to achieve free in‐plane folding–unfolding to meet the requirements of miniaturization and portability of modern electronics?

Herein, we report a material architecture for creating flexible conducting film that is capable of reversible in‐plane folding–unfolding. The soft and highly deformable material is composed of a conductive ionic liquid (IL, i.e., 1‐ethyl‐3‐methylimidazolium bis(trifluoromethylsulfonyl)imide ([EMIm][NTf**_2_**])) embedded in a porous poly(vinylidene fluoride‐*co*‐hexafluoropropylene) (PVDF‐HFP) nanofibrous network with paralleled polylactic acid (PLA) microstructured frames.

## 2 Results and Discussion

### 2.1 Design of Conducting Film with Reversible In‐Plane Folding–Unfolding Function

**Figure 1:**
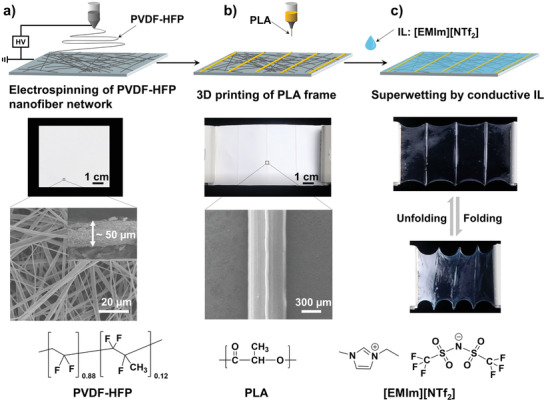
Design of conducting film with reversible in‐plane folding–unfolding function. a) A PVDF‐HFP nanofiber network with thickness of about 50 µm is fabricated by electrospinning. b) PLA frames with width of about 500 µm are introduced onto the PVDF‐HFP nanofiber network by 3D printing. c) The fabricated hierarchical polymer film can be completely wetted by [EMIm][NTf_2_] within 60 s, which shows reversible in‐plane folding–unfolding function.

First, we utilize electrospinning to fabricate a 50 µm thick porous PVDF‐HFP membrane with fibers diameter of about 300 nm (**Figure** **1**a and Figure S1, Supporting Information). Then, paralleled PLA frames with width of about 500 µm are generated by using 3D printing (Figure 1b). This multistructured film can be completely wetted by [EMIm][NTf_2_] (Figure S2, Supporting Information), and the resulted composite film shows perfect reversible in‐plane folding–unfolding performance (Figure 1c). In our design, the PVDF‐HFP polymer chains can form strong ion–dipole interaction with [EMIm][NTf_2_],^[^ ^2^, ^13^ ^]^ which in turn endowing the nanostructured PVDF‐HFP network with the capability of efficiently holding [EMIm][NTf_2_] to form a liquid layer with the assistance of capillary effect. From the energetic point view, the liquid interface prefers to adopt in‐plane deformation^[^ ^14^ ^]^ with both wrinkled and stacked regions at the microscale (Figure S4 and Movie S1, Supporting Information). Meanwhile, the microstructured PLA frames can resist the shrinkage perpendicular to the folding direction, and thus is favorable for sufficient folding.

### 2.2 Evaluation of In‐Plane Folding–Unfolding Property of the IL‐Infused Electrospun PVDF‐HFP Film

**Figure 2:**
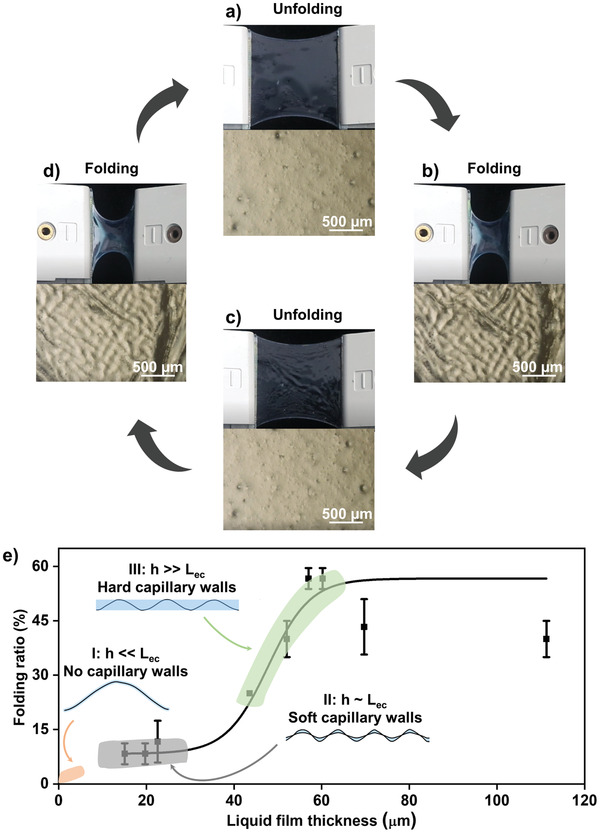
Evaluation of in‐plane folding–unfolding property of the IL‐infused electrospun PVDF‐HFP film. a–d) Macro‐ and micromorphology of the film during reversible folding–unfolding process. e) Effect of liquid film thickness (*h*) on folding behavior. When liquid film thickness is much smaller than the elastocapillary length (*h* << *L*_ec_), no capillary walls can be formed and the folding process is energy‐unfavorable. For *h* ∼ *L*_ec_, soft capillary walls are formed to generate limited folding ratio. For *h* >> *L*_ec_, hard capillary walls are formed and the folding process becomes energy‐favorable. Further increasing the liquid film thickness would lead to an unstable composite film, and thus even a slight decrease of folding ratio. Thus, an optimal liquid film thickness of about 60 µm is chosen for further studies.

We first evaluate the in‐plane folding–unfolding performance of the designed film without PLA frames. Owing to the ion–dipole interaction between PVDF‐HFP polymer chains and [EMIm][NTf_2_], the electrospun PVDF‐HFP membrane can be completely wetted by [EMIm][NTf_2_]. The wetted nanostructured PVDF‐HFP membrane can hold a stable liquid layer due to capillary effect. The generated composite film becomes transparent and macroscopically homogenous with no obvious features at the microscale (**Figure** **2**a and Figure S3, Supporting Information). During compression, the film remains under tension because of the liquid surface tension. The excess film is stored inside the winkled and stacked regions (Figure 2b and Figure S4, Supporting Information). These winkled and stacked regions afterward disappear when the external compression is released, and the film recovers to its initial state (Figure 2c). This folding–unfolding process is perfectly reversible (Figure 2a–d). The [EMIm][NTf_2_]‐infused PVDF‐HFP composite film is quite stable at different folding ratios. The composite film can achieve reversible in‐plane folding–unfolding for at least 20 000 cycles, and the corresponding conductivity varies slightly (Figure S5, Supporting Information), which is probably owing to its relative uniform microstructures at different folding ratios (Figure S6, Supporting Information). To confirm the importance of the capillary effect between the PVDF‐HFP nanofibers and [EMIm][NTf_2_] for reversible folding–unfolding performance, we also evaluate other three films: nanostructured electrospun PVDF‐HFP membrane without ILs, casting PVDF‐HFP membrane wetted by ILs, and casting PVDF‐HFP membrane without ILs. None of the three possesses in‐plane folding–unfolding property (Figure S7, Movie S2, Supporting Information).

It should be noted that the liquid film thickness (*h*), which can be measured by a colorimetry method (Figure S8, Supporting Information), is a key parameter to affect the folding behavior of the film. To make clear the underlying mechanism, we analyze the total energy of the system during folding–unfolding process, which consists of elastic energy of the film (*E*_el_) and the surface energy of the liquid/air interface (*E*_su_) under the assumption of constant liquid film volume and free contour length.^[^ ^14^ ^]^ The ratio of *E*_su_/*E*_el_ is proportional to (*h*/*L*_ec_)^2^, where *L*_ec_ is the elastocapillary length.^[^ ^15^ ^]^ In our case, the *L*_ec_ is estimated to be about 10 µm (for details, see the Supporting Information). For *h* << *L*_ec_, *E*_el_ (Section S7 in the Supporting Information) dominants the total energy of the system, and folding the film leads to increased total energy, which means that folding is an energy‐unfavorable process. In this case, no capillary walls formed between the PVDF‐HFP nanofibers and the infused IL and the film is hardly to fold (orange part in Figure 2e). For *h* ∼ *L*_ec_, *E*_su_ is comparable to *E*_el_, and there is a balance between them to minimize the total energy of the system. Soft capillary walls can be formed between the PVDF‐HFP nanofibers and the infused IL to generate limited folding ratio (gray part in Figure 2e). For *h* >> *L*_ec_, *E*_su_ becomes the dominant one, and folding the film apparently decreases the total energy of the system, indicating an energy‐favorable process with effective formation of hard capillary walls between the PVDF‐HFP nanofibers and the infused IL (green part in Figure 2e). However, it is not the case that the higher the liquid film thickness, the larger the folding ratio. A maximum folding ratio of about 57% is achieved with liquid film thickness of about 60 µm. Because there is a saturated liquid film thickness for the electrospun PVDF‐HFP membrane. Further increasing the liquid film thickness leads to an unstable composite film, and thus even a slight decrease of folding ratio (Figure 2e). Therefore, we choose liquid film thickness of 60 µm as the optimal condition for further studies.

### 2.3 Engineering Microstructured Frames to Improve In‐Plane Folding–Unfolding Performance

**Figure 3:**
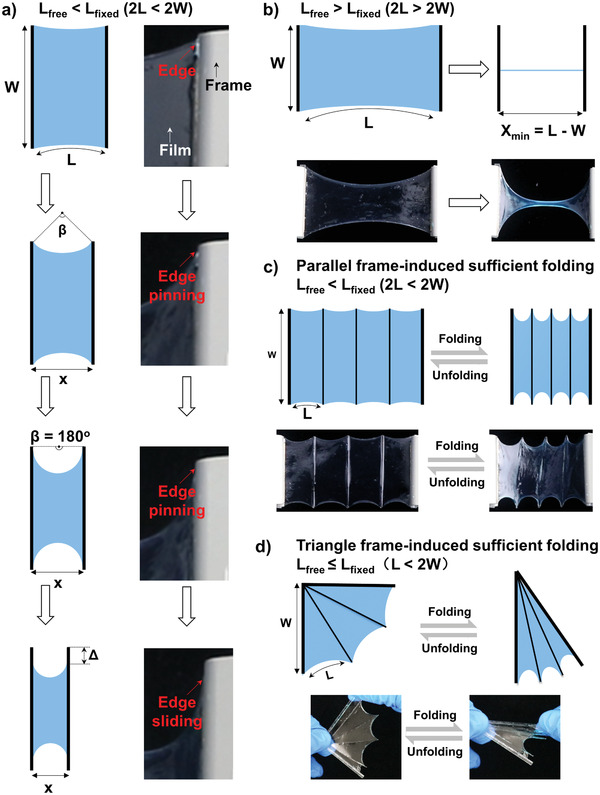
Engineering microstructured frames to improve in‐plane folding–unfolding performance. a) Dynamics during folding process. A force vertical to the folding direction makes the free edges adopt a shape of circular arc with central angle of *β*. Folding the film leads to an increase of *β* until 180°, after which the edge will slide along the fixed support. b) When the total length of free edges (*L*_free_ = 2*L*) is larger than that of fixed edges (*L*_fixed_ = 2*W*) (2*L* > 2*W*), the film can only achieve insufficient folding and eventually turns into a line. c) Introduction of parallel frames satisfying the requirement of 2*L* < 2*W* can ensure sufficient folding. d) Introducing triangle frames is a reliable strategy to achieve sufficient in‐plane folding because the total length of free edges is always smaller than that of the fixed edges (*L* < 2*W*).

Besides the liquid film thickness, the original size of the film also significantly affects the folding behavior. We take a rectangular film of initial length *L* and width *W* as a typical model, attached to two parallel supports. We define the total length of the fixed supports as *L*_fixed_ = 2*W*, while the total length of free edges as *L*_free_ = 2*L*. We first monitor the folding process of the film under the condition of *L*_free_ < *L*_fixed_ (2*L* < 2*W*). As we mentioned above, when satisfying the requirement of *h* >> *L*_ec_, surface energy of the liquid/air interface dominates the total energy of the system. Thus, any way to reduce the surface area of the film will make the system more stable. For our model, there would be a force vertical to the folding direction to minimize the surface area of the film along with the folding process, which makes the free edges adopt a shape of circular arc with central angle of *β*. As shown in **Figure** **3**a, during the initial folding process, *β* increase quickly from a value close to zero until to a maximum value of 180° to minimize the surface area of the film. After that, *β* keeps constant and instead the free edge slides along the fixed support to further minimize the total energy of the system. This folding character is not a problem for *L*_free_ < *L*_fixed_ (Movie S3, Supporting Information). However, it is indeed a big problem for *L*_free_ > *L*_fixed_, where the film gradually turns to a line with continuous folding (Figure 3b and Movie S3, Supporting Information). Further folding will lead to failure of in‐plane shrinkage owing to the gravity of the aggregated film, which means that we can only achieve insufficient folding under this condition. Fortunately, we find a way to solve this problem by introducing paralleled microstructured frames. Specifically, we introduce paralleled microscaled PLA frames onto the porous PVDF‐HFP membrane before wetting by [EMIm][NTf_2_]. Through this strategy, we are able to let *L*_free_ < *L*_fixed_, thus achieving sufficient folding (Figure 3c and Movie S3, Supporting Information). Furthermore, we find that introduction of triangular frames is more robust to gain sufficient in‐plane folding property, in which case the length of free edges (*L*_free_, *L*) is always smaller than that of the fixed edges (*L*_fixed_, 2*W*) (Figure 3d and Movie S3, Supporting Information). In fact, we can find similar examples in nature. There exist triangular skeletons in the wings of bats,^[^ ^16^ ^]^ which help them to reversibly fold–unfold their wings in large extend. Another example is the design of Chinese fan with triangular frames. Overall, we have carefully studied the folding process of our designed films, and realized that introduction of microstructured frames is an effective way to achieve sufficient folding. In contrast to the reported stretchable conducting films,^[^ ^17^ ^]^ the conductive PVDF‐HFP/IL composite film shows distinct in‐plane folding property under compression.

### 2.4 Fabrication of Highly Conductive PVDF‐HFP/PEDOT:PSS/IL Composite Film with Reversible In‐Plane Folding–Unfolding Performance

**Figure 4:**
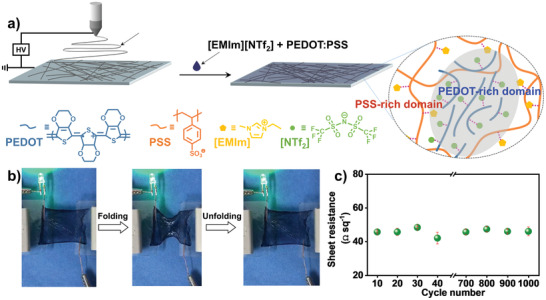
Fabrication of highly conductive PVDF‐HFP/PEDOT:PSS/IL composite film with reversible in‐plane folding–unfolding performance. a) Preparation of highly conductive composite film by wetting the electrospun PVDF‐HFP film with mixture of PEDOT:PSS and ILs. b) The highly conductive composite film can light an LED, and the LED brightness keeps almost unchanged during the folding–unfolding processes. c) Sheet resistance of the film only fluctuates in a relatively small range, between 40 and 50 Ω sq^−1^, during 1000 cycles of in‐plane folding–unfolding.

For practical applications, besides the large deformation, conductivity of the films should also be considered. In our case, conductivity of the fabricated film originating from the infused IL is about 1 S m^−1^ close to that of neat IL, with sheet resistance in the order of 4–5 × 10^3^ Ω sq^−1^. However, we can improve the conductivity of the film to a high level by tuning the composition of the infused conductive liquids.^[^ ^18^ ^]^ As a demonstration, we introduce a conductive mixture containing [EMIm][NTf_2_], poly(3,4‐ethylenedioxythiophene):polystyrene sulfonate (PEDOT:PSS, 1:2, volume ratio), and Capstone FS‐30 as the surfactant (0.1 vol% to [EMIm][NTf_2_]) into the porous PVDF‐HFP membrane, followed by curing at 80 °C for 15 min, to generate highly conductive films with sheet resistance lowering by two orders. The improved conductivity ascribes to the strong electrostatic interactions between [EMIm][NTf_2_] and PEDOT:PSS, leading to separated microstructures with conductive PEDOT‐rich domains and insulating PSS‐domains (**Figure** **4**a). More importantly, the high conductivity of the film remains during the in‐plane folding–unfolding process. As shown in Figure 4b, brightness of a light‐emitting diode (LED) light is unchanged during the folding–unfolding process (Movie S4, Supporting Information). Sheet resistance of the film only fluctuates in a relatively small range, between 40 and 50 Ω sq^−1^ (Figure 4c). We can probably further improve the conductivity by using other conductive liquids such as liquid metals,^[^ ^19^ ^]^ although there may be some issues to be addressed currently. Anyway, we are able to tune the conductivity of the film to a very high level, which would further open up new doors for the applications of this kind of new materials.

## 3 Conclusion

In conclusion, we have developed a method relying on conductive liquids‐infused multistructured membrane to prepare flexible conducting films with large deformable in‐plane folding–unfolding property. On the one hand, molecular‐scale interaction between the polymer chains and infused liquids endows the nanostructured membrane with superwettability, which further provides the driving force—capillary force—to achieve reversible in‐plane folding–unfolding. From this point of view, our method should be broadly applicable to various systems by tuning multi‐interactions (e.g., hydrogen bonding, electrostatic interaction, *π*–*π* stacking, coordination) between different nanostructured networks and different liquids. The infused liquids are not limited to conductive liquids. They can also be liquid crystal, magnetic fluids, or ferroelectric fluids, and thus many other functional even responsive materials would be possible. On the other hand, rational design of the microscaled frames makes it possible to realize enhanced folding–unfolding property. In the present demonstration, we just use frames with width of hundreds of micrometers to confirm the feasibility. For applications requiring higher resolution, frames with smaller sizes could be made compatible with photolithography and other microfabrication techniques. We anticipate our work to provide an approach in the fabrication of flexible materials with desirable functions.

## Conflict of Interest

The authors declare no conflict of interest.

## Supporting information

Supporting Information

Supplemental Movie 1

Supplemental Movie 2

Supplemental Movie 3

Supplemental Movie 4

## Data Availability

Research data are not shared.
