# Supplementary material for: Flexible Conducting Composite Film with Reversible In‐Plane Folding–Unfolding Property
Source: Adv Sci (Weinh). 2021 Aug 13;8(20):2102314. doi: 10.1002/advs.202102314 (PMC8529486; doi:10.1002/advs.202102314)
Supplement: Supplementary file 1 — Supporting Information [file ADVS-8-2102314-s002.pdf]

© 2021 Wiley-VCH GmbH

ADVANCED SCIENCE Open Access

Supporting Information

for *Adv. Sci.,* DOI: 10.1002/advs.202102314

Flexible conducting composite film with reversible in-plane folding-unfolding property

Peiru Sun, Chuao Ma Yong Chen,* and Hongliang Liu,*

Supplementary Information

Flexible conducting composite film with reversible in-plane folding-unfolding property

Peiru Sun, Chuao Ma Yong Chen,* and Hongliang Liu,*

P. Sun, Prof. H. Liu

School of Chemistry and Chemical Engineering, Yantai University, Yantai 264005, P. R. China. E-mail: liuhl@mail.ipc.ac.cn.

P. Sun, Prof. Y. Chen

School of Metallurgy and Materials Engineering, Chongqing University of Science and Technology, Chongqing 401331, P. R. China. E-mail: yongchen998@163.com

C. Ma

College of Chemistry, Jilin University, Changchun 130012, P. R. China

Corresponding author's email address: liuhl@mail.ipc.ac.cn

1. Materials: Poly (vinylidene fluoride-co-hexafluoropropylene) ($PVDF-HFP_{8812}$) (M_w = 4.0×10^5) was purchased from Sigma-Aldrich. Anhydrous acetone was purchased from Beijing Chemical Works (China). Polylactic acid (PLA) was purchased from Shenzhen Creality 3D Technology Co.,LTD. 1-ethyl-3-methylimidazolium bis((trifluoromethyl)sulfonyl)imide([EMIm][NTf_2]) was purchased from Lanzhou Yulu Fine Chemical Co.,Ltd.(China). Fluorosurfactant (Capstone FS-30) were purchased from DuPont. Poly(3,4-ethylenedioxythiophene)-poly (styrenesulfonate) (PEDOT: PSS, PH1000) was purchased from Clevios. All the reagents were used without further modification unless specially mentioned.

2. Fabrication of flexible conducting composite film with reversible in-plane folding-unfolding character: The composite film was fabricated through three steps. Firstly, porous PVDF-HFP membrane was prepared by an electrospinning method. Typically, solid PVDF-HFP powder (5 g) and acetone (50 ml) were heated to 60°C and stirred for 2 h for complete dissolution. Then the solution was electrospun onto an aluminium foil through a syringe equipped with a Langer pump at ambient conditions. The distance between the syringe tip and the aluminium foil was 15 cm, electrospinning voltage was 25 KV, and the injection speed was 5 ml min^{-1}. After 15 min, a 50 μm-thick porous PVDF-HFP nanofibrous membrane was generated. The average diameter of the PVDF-HFP nanofibers was 300 ± 160 nm. Secondly, PLA frames with diameters of about 300 nm and spacing about 2.5 cm were prepared by using a 3D printing machine. The nozzle diameter is fixed to 200 μm. Thirdly, the multi-structured film was peeled off from the aluminium foil and cut into rectangle shape (1.7 cm × 2 cm). Then the film was fully wetted by 8 μl [EMIm][NTf_2] to form conducting composite film.

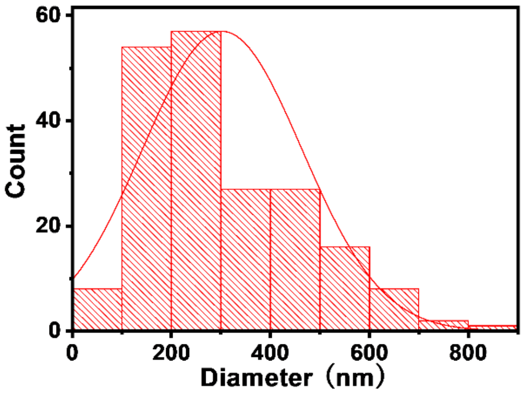

Figure S1. Diameters distribution of the PVDF-HFP nanofibers. The average diameter is 300 ± 160 nm.

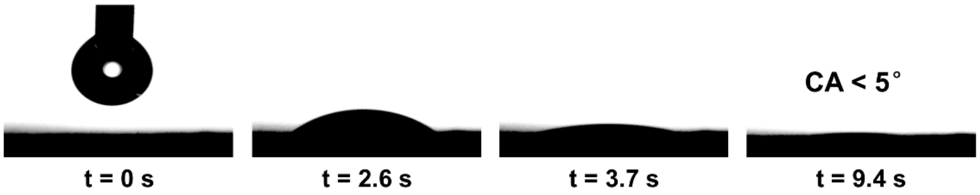

Figure S2. The PVDF-HFP nanofiber network can be easily wetted by a 2 μL [EMIm][NTf_2] droplet within 9.4 s with contact angle lower than 5°.

3. Optical transparency of PVDF-HFP membrane and PVDF-HFP composite film wetted by [EMIm][NTf_2]: UV-Vis spectrophotometer was performed to measure the transparency of PVDF-HFP membrane and PVDF-HFP/IL composite film. The optical transmittance of PVDF-HFP/IL composite film can exceed 80% in the visible light range (Figure S3, black curve). By contrast, the maximum optical transmittance of PVDF-HFP membrane is less than 15% (Figure S3, red curve). The inserted digital photos can also prove that the PVDF-HFP/IL composite film has a higher optical transmittance.

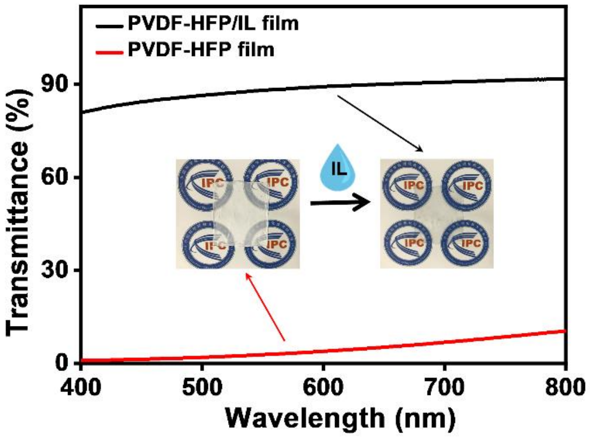

Figure S3. Optical transmittance of the PVDF-HFP membrane and the PVDF-HFP/IL composite film in the visible light range.

4. Morphology of the PVDF-HFP/IL composite film during folding-unfolding: During compression, the PVDF-HFP/IL composite film appears wrinkled and stacked regions at the micro scale. These wrinkled and stacked regions can store excess film and disappear when the compression is released.

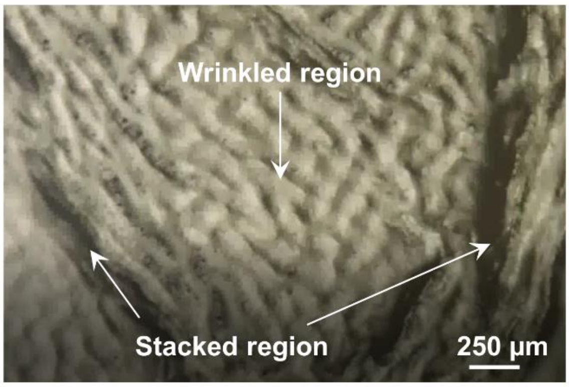

Figure S4. The PVDF-HFP/IL composite film prefers to adopt in-plane deformation with both wrinkled and stacked regions at the micro scale.

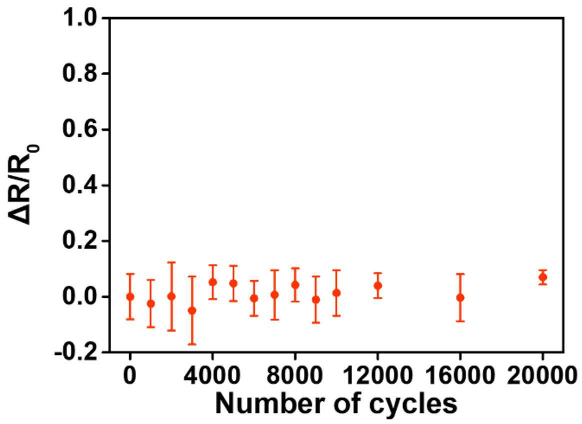

Figure S5. Conductivity changes of the composite film during 20000 cycles of in-plane folding-unfolding. The maximum folding ratio is 50%.

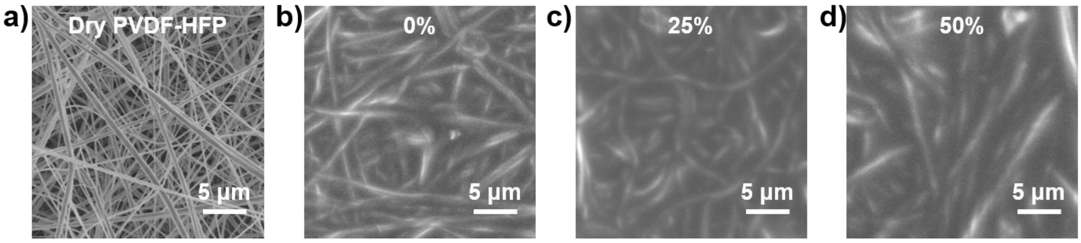

Figure S6. SEM images of (a) the dry PVDF-HFP membrane without [EMIm][NTf_2] and (b-d) [EMIm][NTf_2]-infused composite film at different folding ratios (0% means the initial state).

5. Importance of the capillary effect for reversible in-plane folding-unfolding property: We fabricated four types of films: nanostructured electrospun PVDF-HFP membrane wetted by ILs, nanostructured electrospun PVDF-HFP membrane without ILs, casting PVDF-HFP membrane wetted by ILs, and casting PVDF-HFP membrane without ILs. Only in the case of nanostructured electrospun PVDF-HFP membrane wetted by ILs, the film exhibits reversible folding-unfolding performance. The other three films show spatial dislocations out-of-plane when compressed.

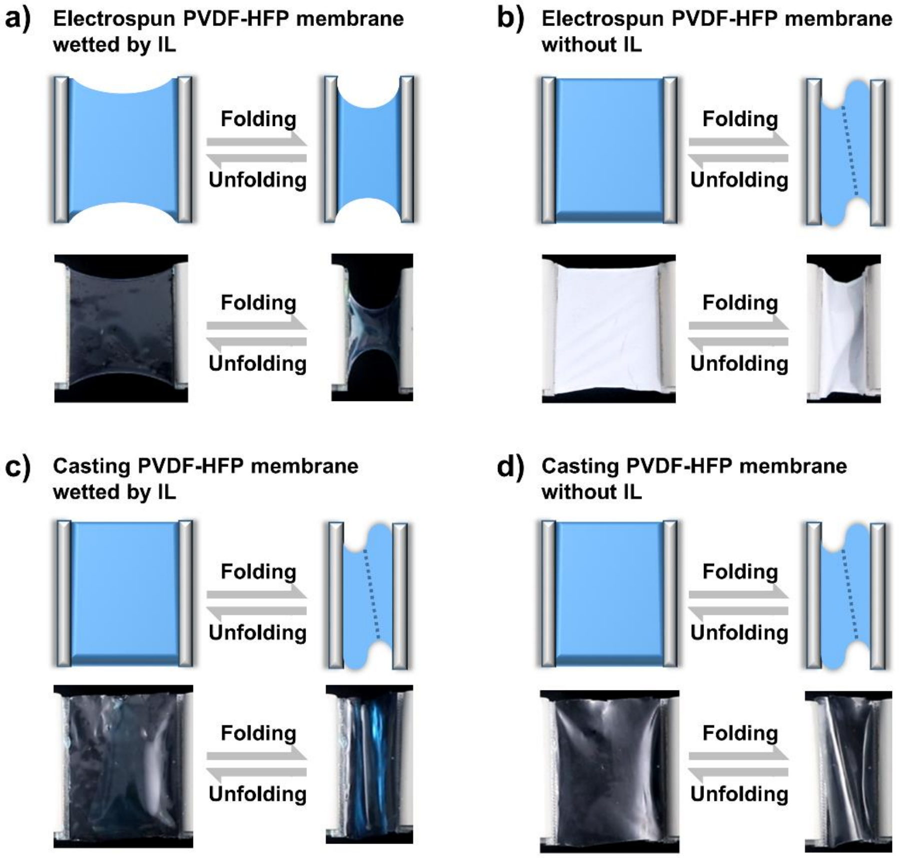

Figure S7. Influence of capillary effect on reversible in-plane folding-unfolding performance. The nanostructured electrospun PVDF-HFP membrane wetted by [EMIm][NTf_2] demonstrates reversible in-plane folding-unfolding performance because of the existence of capillarity (a). While films without capillarity owing to lack of infusing liquid (b), nanostructures (c), or both (d) do not possess the function of reversible in-plane folding-unfolding.

6. Measurement of the liquid film thickness: We measured liquid film thickness of the IL-infused membrane using a colorimetry method. First, we placed a coverslip between two clean slides to create a slope and secure the ends with clip. Then, an IL ([EMIm][NTf_2]) stained with methyl blue was injected between the two slides. Finally, a photograph was taken with a camera when the colour depth between the two slides no longer changed. The photograph analysis is performed using the image processing package Image J software. Grey level can be obtained from the colour depth of the blue IL on the photograph through Image J software. The

results of the standard curve show that as the liquid thickness increases, the grey level decreases (Figure S8). Therefore, comparing the photograph's grey level on the membrane and the thickness versus the grey level curve, we can estimate the thickness of the liquid film.

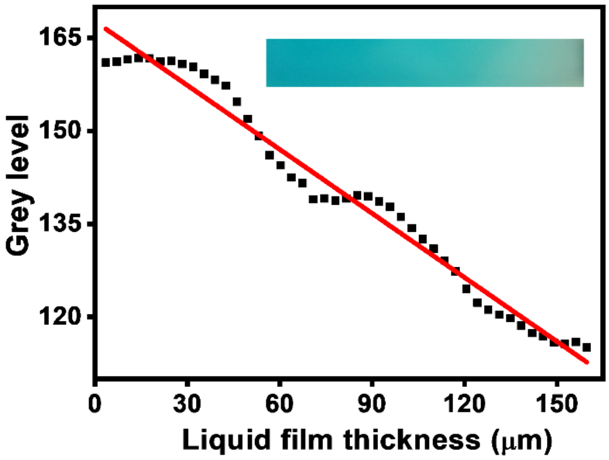

Figure S8. Standard curve of grey level versus liquid film thickness.

7. Calculation of elastocapillary length L_{ec}: L_{ec} is calculated by using the following equation[1]:

$$L_{ec} = \sqrt{B / \gamma} \tag{S1}$$

where γ is the surface tension of the liquid (γ = 36 mN/m for [EMIm][NTf_2]), and B is the membrane bending rigidity per unit depth. B can be roughly estimated as:

$$B = \alpha \frac{t_0}{a} E a^3 \tag{S2}$$

where t_0 is the dry thickness of the membrane (t_0 = 50 μm), a is radius of the fiber composing the membrane (a is in the range of 100-300 nm), E is the PVDF-HFP Young's modulus (E = 3 GPa, Fig. S9), and α is a dimensionless parameter accounting for the membrane porosity ($\alpha = 2 \times 10^{-4}$). According to equations (S1) and (S2), L_{ec} is estimated to be about 10 μm.

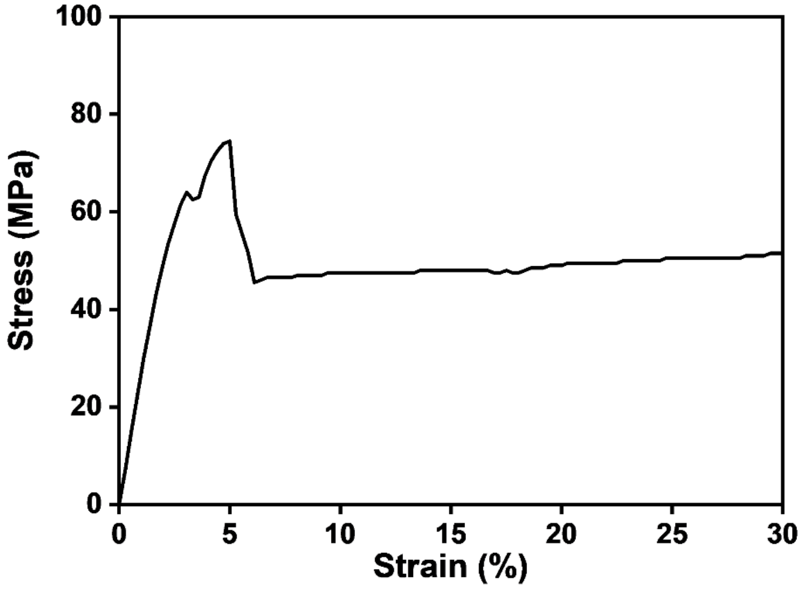

Figure S9. Tensile stress–strain curve of the PVDF-HFP film at 50 mm min^{-1} stretching rate. The initial sizes of the film are length of 1.5 cm, width of 4 mm and thickness of 25 μm.

Captions for Movies S1 to S4

Movie S1. Appearance of both wrinkled and stacked regions at the micro scale during reversible in-plane folding-unfolding.

Movie S2. Dynamic folding-unfolding processes of four different kinds of films. **Movie S3.** Effect of original size on the folding-unfolding behaviour.

Movie S4. Highly conductive PVDF-HFP/PEDOT:PSS/IL composite film can light a LED during in-plane folding-unfolding process.

References:
[1] P. Grandgeorge, N. Krins, A. Hourlier-Fargette, C. Laberty-Robert, S. Neukirch, A. Antkowiak, *Science* 2018, **360**, 296.
